# Oncogenic zinc finger protein ZNF687 accelerates lung adenocarcinoma cell proliferation and tumor progression by activating the PI3K/AKT signaling pathway

**DOI:** 10.1111/1759-7714.14856

**Published:** 2023-03-21

**Authors:** Mingchun Li, Zhihua Liu, Zan Hou, Xiangcai Wang, Huaqiu Shi, Yamei Li, Xuewen Xiao, Zhixian Tang, Jianqiong Yang, Yaoling Luo, Minhong Zhang, Ming Chen

**Affiliations:** ^1^ Department of Radiation Oncology, The Second Affiliated Hospital Soochow University Suzhou China; ^2^ Department of Oncology, The First Affiliated Hospital Gannan Medical University Ganzhou China; ^3^ The Clinical Medicine Research Center, The First Affiliated Hospital Gannan Medical University Ganzhou China; ^4^ Department of Pathology, Guangdong General Hospital Guangdong Academy of Medical Sciences Guangzhou China; ^5^ Department of Radiation Oncology Sun Yat‐sen University Cancer Center, State Key Laboratory of Oncology in South China, Collaborative Innovation Center of Cancer Medicine Guangzhou China; ^6^ Department of Pathology, The First Affiliated Hospital Gannan Medical University Ganzhou China; ^7^ Department of Cardiothoracic Surgery, The First Affiliated Hospital Gannan Medical University Ganzhou China; ^8^ Department of Radiation Oncology, Sun Yat‐sen University Cancer Center, State Key Laboratory of Oncology in South China, Collaborative Innovation Center for Cancer Medicine Sun Yat‐sen University Guangzhou China

**Keywords:** cell cycle, EMT, lung adenocarcinoma, PI3K/ AKT, ZNF687

## Abstract

**Background:**

Zinc finger protein 687 (ZNF687) has previously been discovered as a potential oncogene in individuals with giant cell tumors of the bone, acute myeloid leukemia, and hepatocellular carcinoma. However, its role and mechanism in lung adenocarcinoma (LUAD) remain unclear.

**Methods:**

In LUAD cells, tumor, and matched adjacent tissue specimens, quantitative real‐time RT‐ polymerase chain reaction (qRT‐PCR), western blotting analyses, and immunohistochemistry staining (IHC) were conducted. Cell counting kit‐8 (CCK8) assay, clonogenicity analysis, flow cytometry, and transwell assays were utilized to detect ZNF687 overexpression and knockdown impacts on cell growth, colony formation, cell cycle, migration, and invasion. Bioinformatic studies, qRT‐PCR and western blotting studies were employed to validate the underlying mechanisms and signaling pathways implicated in the oncogenic effect of ZNF687.

**Results:**

This study demonstrated that ZNF687 expression was elevated in LUAD cells and tissues. Individuals with upregulated ZNF687 had a poorer prognosis than those with downregulatedZNF687 (*p* < 0.001). ZNF687 overexpression enhanced LUAD growth, migration, invasion and colony formation, and the cell cycle G1‐S transition; additionally, it promoted the epithelial‐mesenchymal transition (EMT). In contrast, knocking down ZNF687 showed to have the opposite impact. Moreover, these effects were associated with the activity of the phosphatidylinositol 3‐kinase (PI3K)/protein kinase B (AKT) signaling mechanism.

**Conclusion:**

ZNF687 was upregulated in LUAD, and high ZNF687 expression levels are associated with poor prognoses. The activation of the PI3K/AKT signaling pathway by upregulated ZNF687 increased the proliferation of LUAD cells and tumor progression. ZNF687 may be a beneficial predictive marker and a therapeutic target in LUAD.

## Graphical abstract

By suppressing the PI3K/AKT signaling pathway, ZNF687 knockdown may lower the growth, invasion, and migration of LUAD cells.

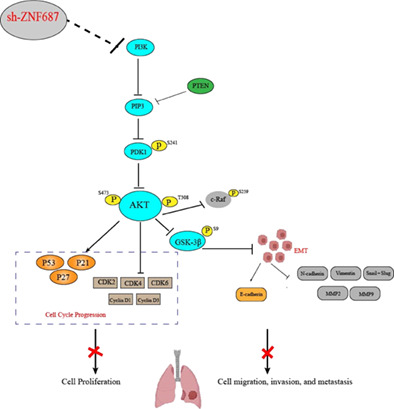

## INTRODUCTION

Lung cancer is the main reason for morbidity and mortality in China and worldwide. Non‐small cell lung cancer (NSCLC) represents 80%–85% of lung tumor cases, and approximately 20% of patients with NSCLC live for at least 5 years with the disease after diagnosis.^1^, ^2^ Lung adenocarcinoma (LUAD) is the most common form of lung cancer.^3^ Distant metastasis and recurrence are important factors affecting individuals with lung cancer prognosis.^4^ Several human cancers, including LUAD, are affected by the dysregulation of gene encoding proteins, recognition of oncogenic pathways, tumor suppressor genes, and other genes associated with lung cancer.^5^ The examination of protein‐coding genes with important regulatory functions is a highly studied field. It is essential to further explore the molecular pathogenesis of protein‐coding gene‐related diseases and to develop new targeted therapies for LUAD.

A large transcriptional regulatory network is regulated by the zinc finger protein 687 (ZNF687), which functions as the central hub and is a recently recognized C2H2 zinc finger protein that binds to ZNF592 and ZNF532 to create a heterotrimeric Z3 complex. According to numerous studies, ZNF687 performs an essential function in the regulation of the epigenome, particularly in the transcriptional silencing of DNA damage in genomic regions.^6^ Recent studies indicate that ZNF687, which is elevated and mutated in giant cell tumors, hepatocellular carcinoma (HCC), acute myeloid leukemia, and bone cancer, has an oncogenic function in cancer.^7^, ^8^, ^9^, ^10^ A previous bioinformatic study revealed that ZNF687 may be highly expressed in LUAD.^11^ We previously found that ZNF687 may act as a transcription factor of oncogene cyclin‐dependent kinase regulatory subunit 2 (CKS2) via informatic analysis and luciferase assay (Li MC, unpublished data). Nevertheless, ZNF687 function and mechanism in LUAD remain unclear. Herein, we preliminarily explored the biological function and related mechanism of ZNF687 in LUAD.

## METHODS

### Tissue microarray

A tissue microarray (microarray no. HLugA180Su07), which contained 98 pairs of LUAD and 82 pairs of paracarcinoma tissue samples, was obtained from Shanghai Outdo Biotech Co., Ltd. All participants experienced surgery without chemotherapy, immunotherapy, targeted therapy, or radiotherapy. In addition, all samples were independently diagnosed by two senior consultant pathologists and followed up regularly until August 2014. As previously described,^12^ the patient characteristics are summarized in Table 3.

### Clinical specimen collection

Eight cases of fresh LUAD tumors and matched adjacent tissue specimens were acquired from the First Affiliated Hospital of Gannan Medical University from January to May 2021. After surgery, tissue specimens were directly frozen in liquid nitrogen and then in −80°C freezers. Furthermore, paraffin‐embedded pathological specimens of 20 paired LUAD and paracarcinoma tissues were collected for immunohistochemistry staining (IHC) analysis. The patients had not been treated with any targeted therapies, immunotherapies, chemotherapy, or radiotherapy.

### Cell culture

A549 and BEAS‐2B cells were acquired from the Type Culture Collection Committee of the Chinese Academy of Sciences cell bank. PC9, HCC827, and H1975 were generously provided by Professor Yamei Chen (Zhejiang Key Laboratory of Radiation Oncology), and H1299 and HBE were supplied by Professor Wenmei Su (Guangdong Medical University). A549 cells were cultured in F12K media (21 127 022; Invitrogen) with 10% fetal bovine serum (FBS), while PC9, HCC827, H1975, H1299, and HBE cells were cultivated in RPMI‐1640 medium (A1049101; Gibco) with 10% FBS (10 099 141; Gibco). At 37°C and 5% CO_2_, all cells were cultivated in a humidified cell incubator.

### IHC assays

The IHC assays were implemented as previously described.^13^, ^14^ All tissue microarrays were dewaxed, hydrated, and microwaved for antigen recovery in citrate buffer (10 mM citric acid, pH 6.0). After being blocked in 5% animal serum, they were treated overnight at 4°C with the primary antibody against ZNF687 (1:100, Cat no. HPA021193, Sigma‐Aldrich). Segments were treated with the secondary antibody (1:1000, Cat no. ab6721, Abcam) for 1 h the following day. A microscope was used to photograph the sections stained with hematoxylin.

IHC staining intensity was calculated:^15^ 0 represented no staining; 1 represented <10%; 2 represented 10%–24%; 3 represented 25%–50%; 4 represented >50% depending on the percentage of the positively stained cells for ZNF687. A score of three or four was considered a high expression for ZNF687. All samples were independently diagnosed by two senior consultant pathologists.

### Vectors, retroviral infection, and transfection

ZNF687 knockdown (sh‐ZNF687#1, sh‐ZNF687#2, and sh‐ZNF687#3) with negative control (sh‐NC), and ZNF687 overexpression (Lv‐ZNF687) with negative control (vector) lentivirus vectors, were constructed and packaged by Shanghai Novobio Biotechnology Company.

A549 and H1299 cells were infected with lentivirus vectors (MOI = 10) as determined by preliminary experiments. The blasticidin S concentration (ant‐bl‐05, Invivogen) was used to further screen and expand for 2 weeks. The sequences for shRNAs were as follows:

shZNF687#1

CACCGCTACTGAGGAGTCGTCTTCACGAATGAAGACGACTCCTCAGTAGC

Sh‐ZNF687#2 CACCGCCCTTGAAGGAAGAAGATGACGAATCATCTTCTTCCTTCAAGGGC

sh‐ZNF687#3

CACCGCGAATGAAGCCATCCATTCTCGAAAGAATGGATGGCTTCATTCGC

shNC

GAAACGATATGGGCTGAATACCGAAGTATTCAGCCCATATCGTTTC

### Cell counting kit‐8 (CCK8) assay

CCK‐8 assay (Beyotime) was employed to determine LUAD cell viability. At 37°C and 5% CO_2_, cells were cultivated in 96‐well plates (2 × 10^3^ cells/well) for 0, 24, 48, and 72 h. Each well was then incubated for 2 h at 37°C with 10 μl of CCK‐8 solution. Lastly, a microplate reader was used to detect 450 nm absorbance.

### Colony formation assays

After LUAD cell transfection according to the above mentioned steps, 400 cells/well were placed in triplicate in six‐well plates for 1 to 2 weeks. Colonies were stained for 15 min with crystal violet solution after being fixed in methanol for 30 min. Counting colonies with 50 or more cells were performed.

### Cell cycle assays

A cell cycle assay kit (Beyotime) was used to treat cells in a good growth stage that had been fixed in 70% cold ethanol. Flow cytometry (BD Biosciences) was utilized to examine the cell cycle distribution.

### Migration and invasion assays

Migration and invasion assays were conducted as previously defined.^16^ Matrigel‐coated transwell chambers were filled with 2 × 10^4^ LUAD cells/well resuspended in serum‐free media (356 234, Corning). Then, 600 μl of media with 15% FBS was introduced to the bottom chamber of the 24‐well plate, and the culture was regularly grown for 24 h. The specimens were subjected for 1 h to 0.1% crystal violet stain (Beyotime), followed by three phosphate buffered saline (PBS) washes. Cells were counted in five regions under a microscope.

The transwell migration assay method was similar to the transwell invasion assay defined above, except that there was no Matrigel coating.

### Quantitative real‐time PCR (qRT‐PCR) analysis

Depending on the manufacturer's instructions, total RNA was acquired from LUAD cell lines utilizing the TRIzol reagent (155 596 026, Invitrogen). A reverse transcription kit (TransGen Biotech) was employed for the reverse transcription reaction to create complementary DNA from 2 μg of total RNA.A 2 × SYBR Green PCR Master Mix (TransGen Biotech) was used for qRT‐PCR. Table S1 lists the primers (Sangon).

### Western blotting analyses

The cell lines were digested, centrifuged, and lysed using protein lysis to obtain total cell proteins. After protein quantification utilizing the BCA protein quantitation assay (KeyGENBioTECH) method, protein electrophoresis was performed using 8%–12% SDS–PAGE, and 20 μg of the specimen was introduced to each well. The proteins were detected using a Chemi Dox XRS chemiluminescence imaging technique (Bio‐Rad). ImageJ program (NIH) was employed to analyze the western blotting results. Table S2 illustrates the antibodies.

### Bioinformatic analyses

The GEPIA2 database (http://gepia2.cancer-pku.cn/) was used to acquire the top 100 ZNF687‐correlated genes from LUAD TCGA tumor and normal tissues. The WebGestalt database (http://www.webgestalt.org/) was used for the ZNF687‐correlated genes to analyze the Kyoto Encyclopedia of Genes and Genomes (KEGG) pathway analysis. Table S3 lists the top 100 ZNF687‐correlated genes.

### Statistical analysis

Data analysis was conducted utilizing GraphPad Prism 8.0. The outcomes are shown as the mean ± SD since each trial was conducted three times. For comparisons between the two groups, independent sample *t* tests were employed, and one‐way analysis of variance (ANOVA) was utilized for comparisons between several groups. Fisher's and Pearson's chi‐square tests were applied to assess the clinicopathological features of the individuals with LUAD and ZNF687 status. The survival curves for the patients were created utilizing the Kaplan–Meier technique, and log‐rank analysis was performed to assess any significant differences. *p*‐values < 0.05 were considered statistically significant.

## RESULTS

### ZNF687 overexpression is associated with poor prognosis in patients with LUAD

**FIGURE 1:**
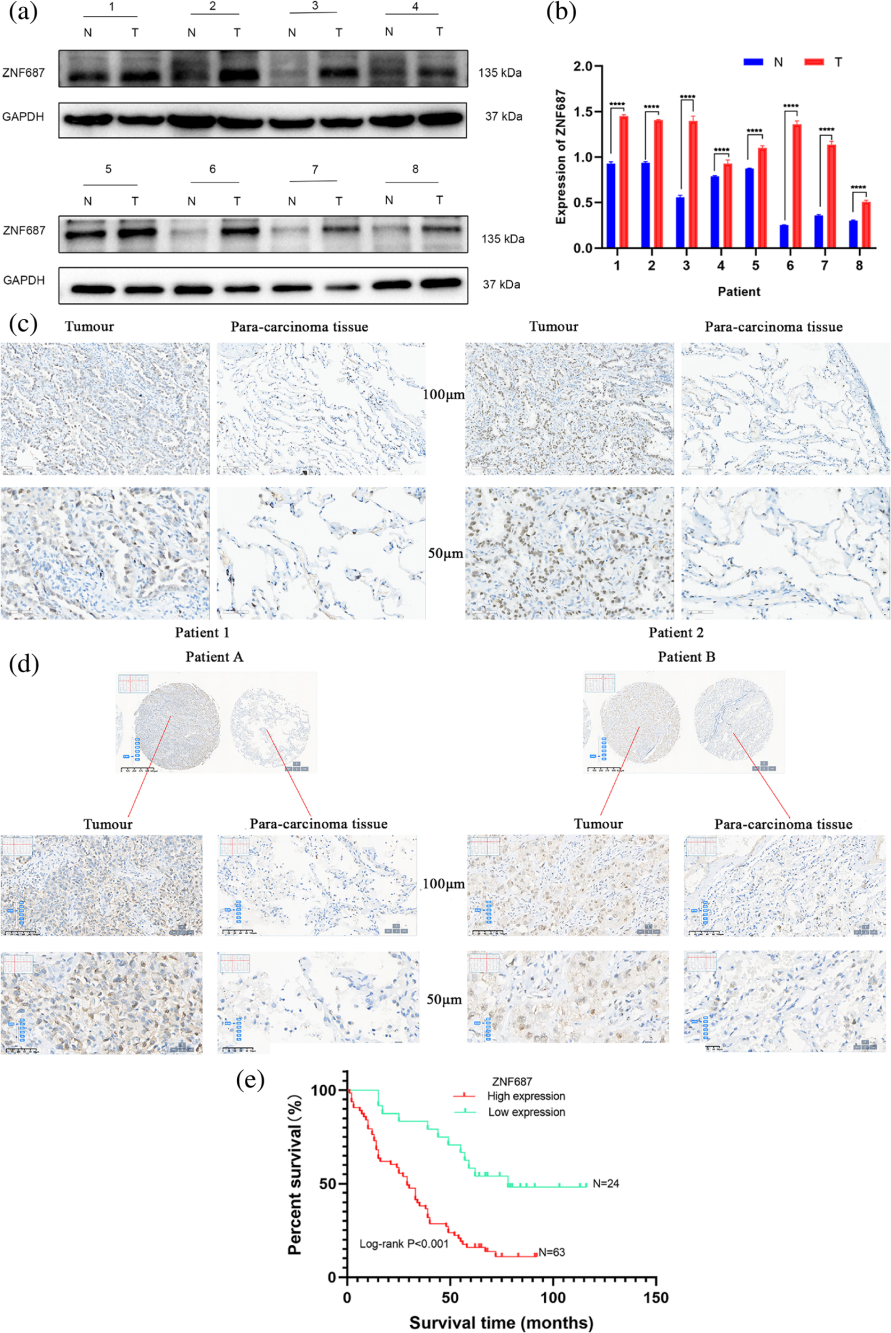
ZNF687 overexpression relates to poor prognosis in lung adenocarcinoma (LUAD) patients. (a, b) ZNF687 protein expression levels were analyzed utilizing western blotting in eight cases of fresh LUAD tumor and matched adjacent tissue specimens. (***p < 0.001, ****p < 0.0001). (c) ZNF687 expression in paraffin‐embedded pathological specimens of 20 paired LUAD and paracarcinoma tissues was identified via immunohistochemistry (IHC) examination (scale bars, 100 and 50 μm, respectively). (d) ZNF687 expression in the tissue microarray, which contained 98 pairs of LUAD samples and 82 pairs of their paracarcinoma tissues detected via IHC analysis (scale bars, 625, 100, and 50 μm, respectively). (e) Kaplan–Meier plotter survival analysis depended on microarray tissue IHC outcomes including 87 patients with LUAD (low vs. high expression; *p* < 0.001).

In our study, western blotting data revealed that ZNF687 protein expression was more in LUAD tissues (T = 8) than in paired paratumor tissues (*N* = 8) (Figures 1a,b). An IHC analysis of 20 paired tumor and paratumor specimens revealed that ZNF687 was strongly expressed in cancer tissues (Figure 1c) (Table 1).

**TABLE 1:** ZNF687 expression sequences in lung adenocarcinoma (LUAD) and matched paratumor tissues were shown in the immunohistochemistry (IHC) analysis.

Sample	ZNF687 expression		Total	*p*‐value (χ^2^)
	High	Low		
	n %	n %		
Tumor	14 (70.0%)	6 (30.0%)	20	0.0002*
Paracarcinoma tissue	2 (10.0%)	18 (90.0%)	20	

IHC staining of ZNF687 in LUAD (*n* = 98) and paratumor tissues (*n* = 82) was performed to explore how ZNF687 expression correlates with clinicopathological features. Compared to paracarcinoma tissues, LUAD tissues had higher ZNF687 expression (Figure 1d) (Table 2). We subsequently analyzed the statistical relationship between ZNF687 and medical features in subjects with LUAD. ZNF687 levels were positively related to clinical characteristics, including lymph node‐positive (*p* = 0.0445), cancer grade (*p* = 0.0293), lymphatic metastasis (*p* = 0.0325), and medical stages (AJCC 7th) (*p* = 0.0253), indicating that ZNF687 overexpression was an independent risk variable for poor outcomes (Table 3).

**TABLE 2:** ZNF687 expression patterns in lung adenocarcinoma (LUAD) and paratumor tissues microarray were revealed in the immunohistochemistry (IHC) analysis.

Sample	ZNF687 expression		Total	*p‐*value (χ^2^)
	High	Low		
	n %	n %		
Tumor	69 (70.4%)	29 (29.6%)	98	
				<0.001*
Paracarcinoma tissue	8 (9.76%)	74 (90.2%)	82	

**TABLE 3:** Association of ZNF687 expression with clinicopathological characteristics of individuals with lung adenocarcinoma (LUAD).

Features	No. of patients	ZNF687 expression		*p‐*value
		High	Low	
All patients	98	69	29	
Age (years)				
≤60	51	34	17	0.5072
>60	47	35	12	
Gender				
Male	55	43	12	0.0749
Female	43	26	17	
Tumor size				
<4 cm	29	13	16	0.3758
≥4 cm	69	39	30	
Lymph node‐positive				
<1	44	26	18	0.0445*
≥1	53	42	11	
Grade				
I	4	4	0	
II	58	35	23	0.0293*
III	35	29	6	
T Infiltrate				
T1–T2	70	48	22	0.4626
T3–T4	26	20	6	
Lymphatic metastasis (N)				
N0	44	26	18	0.0325*
N1–N3	38	31	7	
TNM stage				
I–II	52	31	21	0.0253*
III–IV	45	37	8	
Expression of EGFR (FISH)				
Negative	80	58	22	0.512
Positive	13	8	5	
Expression of ALK (FISH)				
Negative	68	49	19	
Positive	15	10	5	0.5072
PD‐L1				
Positive <5%	32	22	10	
Rate (%) ≥5%	54	37	17	>0.9999
CD8 positive <5%	26	17	9	
Rate (%) ≥5%	46	34	12	0.59

*Note*: Bold values indicate *p* < 0.05.

Abbreviations: EGFR, epidermal growth factor receptor; ALK, anaplastic lymphoma kinase; FISH, fluorescence in situ hybridization; PD‐L1, programmed cell death ligand 1.

In our studies, log‐rank testing and Kaplan–Meier analysis revealed the results depending on microarray tissue IHC outcomes containing 87 individuals with LUAD (there were 98 cases in total, of which 11 cases were lost to follow‐up for survival time) (*p* < 0.001) (Figure 1e).

### ZNF687 protein expression was upregulated in LUAD cell lines

**FIGURE 2:**
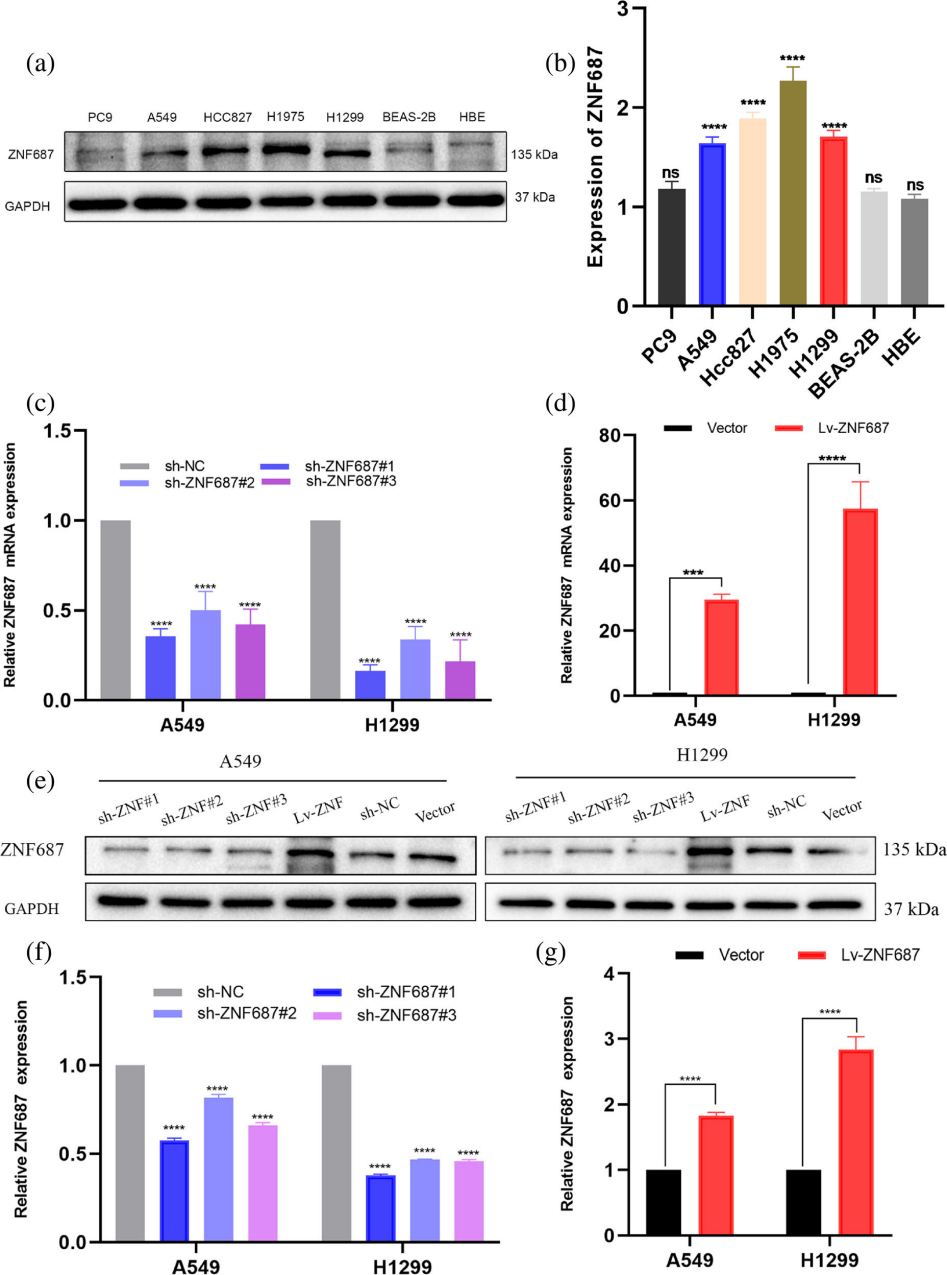
(a, b) ZNF687 expression western blotting analysis in five lung adenocarcinoma (LUAD) cell lines (PC9, A549, HCC827, H1975, and H1299) and noncancerous primary human lung epithelial cells (BEAS‐2B and HBE). (c, d) qRT–PCR and (e, f, g) western blotting validated the efficiency of the enforced ZNF687 overexpression and knockdown efficiency of ZNF687 in A549 and H1299 cells, respectively. *p < 0.05; **p < 0.01; ***p < 0.001; and **** *p* < 0.0001. ns, nonsignificant.

ZNF687 protein levels were identified in two noncancerous primary human lung epithelial cell lines (BEAS‐2B and HBE) and five LUAD cell lines (PC9, A549, HCC827, H1975, and H1299) using western blotting. ZNF687 was highly expressed in A549, HCC827, H1975, and H1299 cells, whereas it was expressed at low levels in PC9, BEAS‐2B, and HBE cell lines (Figures 2a,b).

Huang et al.^17^ screened the shRNA expression vector library, including 3354 shRNA expression vectors covering 2065 genes. ZNF687 was considered a potential p21 suppressor gene, but the mechanism of action remains unclear. A549 (p53‐wild) and H1299 (p53‐null) cells were selected for subsequent evaluations.

The results suggested that ZNF687 was successfully constructed at the protein and mRNA levels (*p* < 0.05). Furthermore, the outcomes exhibited that sh‐ZNF687#1 exhibited the highest knockdown efficiency. Therefore, sh‐ZNF687#1 was chosen as the ZNF687 knockdown cell line sequence to transfect cells for the subsequent experiments (Figures 2c–g).

### ZNF687 increased LUAD cell growth and enhanced cell cycle G1‐S transition in vitro

**FIGURE 3:**
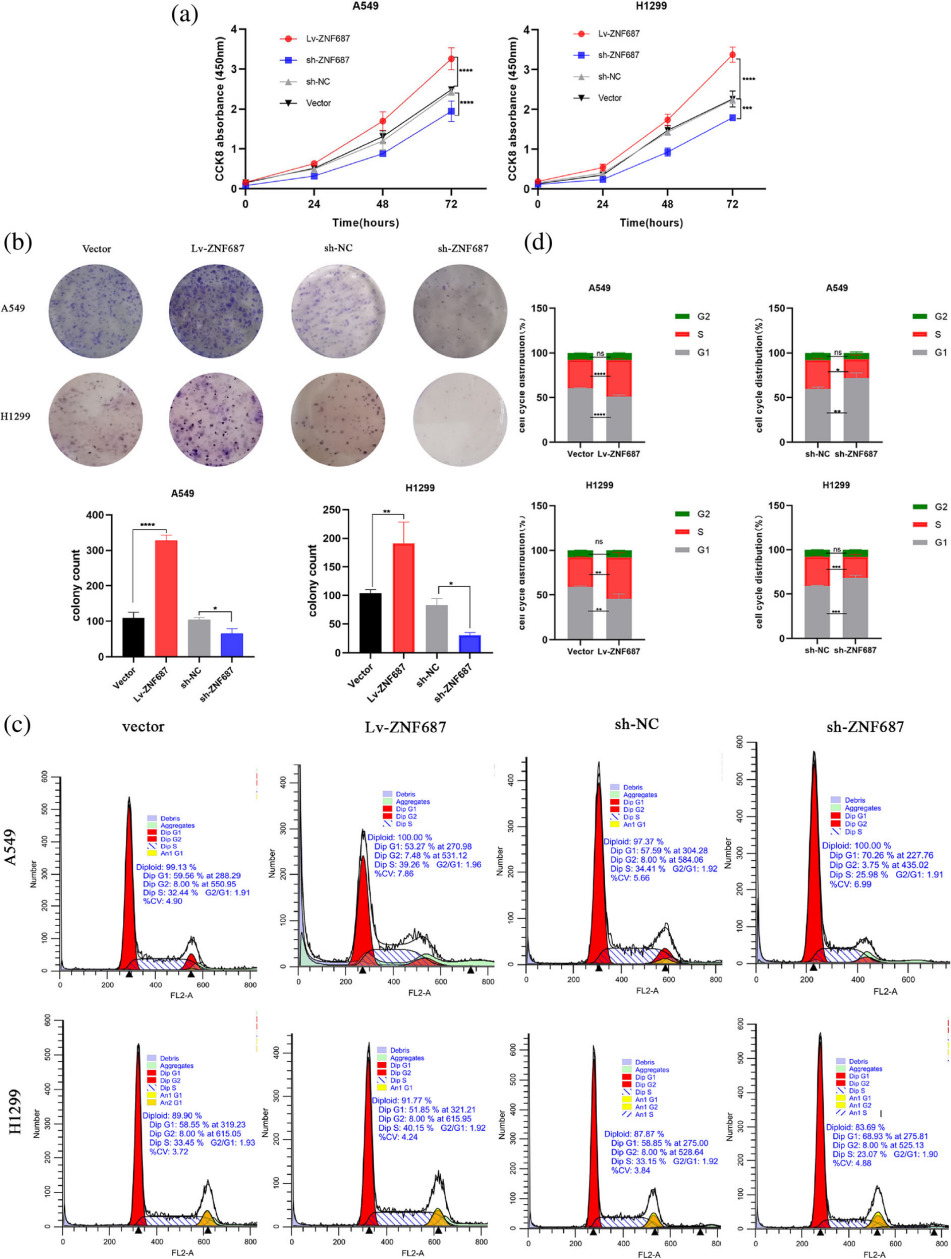
(a) Effect of ZNF687 overexpression and knockdown on cell growth via the cell counting kit‐8 (CCK‐8) assay. (b) A colony formation assay was utilized to assess ZNF687 cell growth. (c, d) Flow cytometry was utilized to identify the distribution of cell cycle phases. *p < 0.05; **p < 0.01; ***p < 0.001; and **** *p* < 0.0001. ns, nonsignificant.

Subsequently, ZNF687 knockdown impact on cell growth was evaluated utilizing the CCK‐8 assay, and ZNF687 knockdown was significantly found to reduce LUAD cell growth, whereas ZNF687 overexpression enhanced proliferation (Figure 3a). Colony formation assays showed that ZNF687 knockdown reduced the colony sizes and numbers of A549 and H1299 cells. Conversely, ZNF687 overexpression in LUAD cells had the opposite effect (Figure 3b).

Flow cytometry was employed to investigate whether ZNF687 may affect cell growth by regulating the cell cycle. Outcomes suggested that ZNF687 knockdown raised LUAD cell proportion in the G1 stage, whereas ZNF687 overexpression reduced them. ZNF687 overexpression elevated the LUAD cell quantity in the S phase. Nevertheless, the percentage of cells in the G2 phase was unaffected (Figures 3c,d). These data indicated that ZNF687 may increase LUAD cell growth and enhance the cell cycle G1‐S transition in vitro.

### ZNF687 regulated cell cycle development by downregulating p21 expression in LUAD cells

**FIGURE 4:**
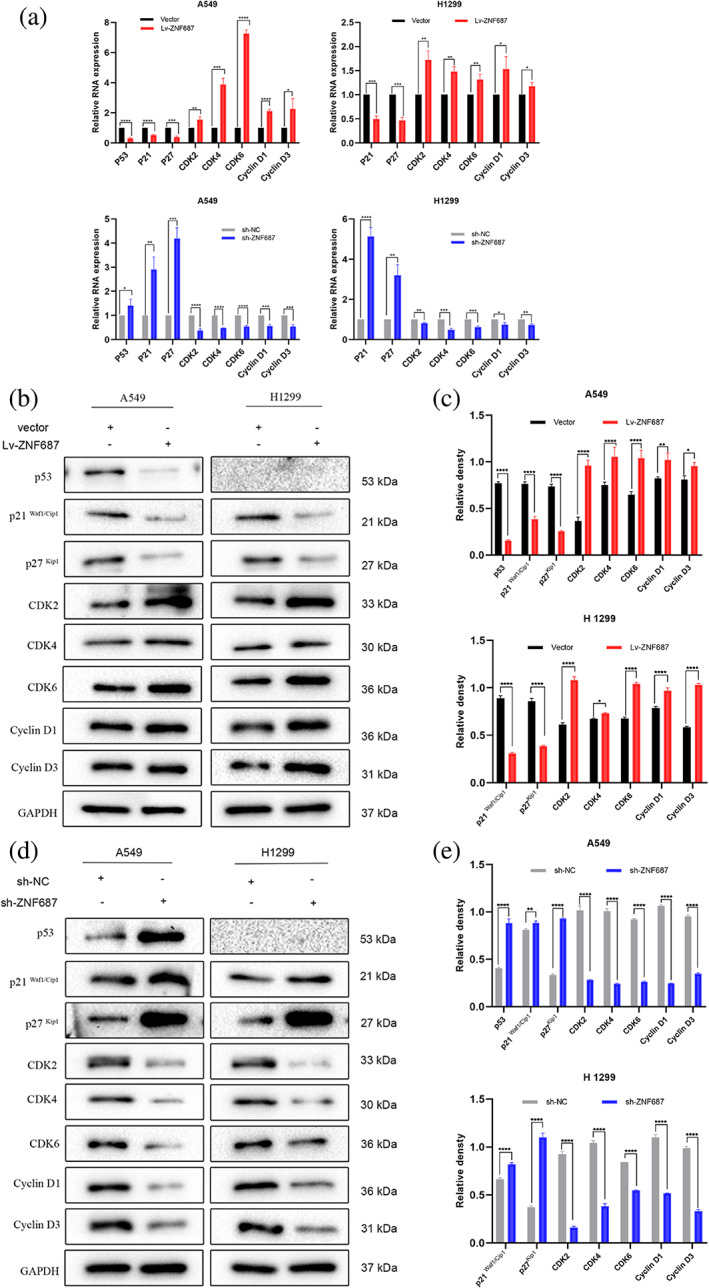
In ZNF687 overexpression or knockdown of LUAD cells, expression of p53, p21, p27, CDK2, CDK4, CDK6, cyclin D1, and cyclin D3 were detected utilizing (a) qRT‐PCR and (b–e) western blot analysis. *p < 0.05; **p < 0.01; ***p < 0.001; and**** *p* < 0.0001. ns, nonsignificant.

To explore the potential pathways by which ZNF687 accelerates tumor growth through modulating cell cycle progression, the protein and mRNA levels of eight cell cycle key genes were examined, including p53, p21, p27, cyclin‐dependent kinase 2 (CDK2), cyclin‐dependent kinase 4 (CDK4), cyclin‐dependent kinase 6 (CDK6), cyclin D1 (CCND1) and cyclin D3 (CCND3). ZNF687 overexpression upregulated the mRNA and protein levels of CDK2, CDK4, CDK6, cyclin D1, and cyclin D3 but reduced p53, p21, and p27 levels, thus revealing that G1/S phase development was stimulated (Figures 4a–g). Conversely, ZNF687 knockdown was observed to have the opposite effect. Therefore, p21 might be up‐ or downregulated in A549 and H1299 cells.

### ZNF687 enhanced the metastasis of LUAD cells through EMT induction

**FIGURE 5:**
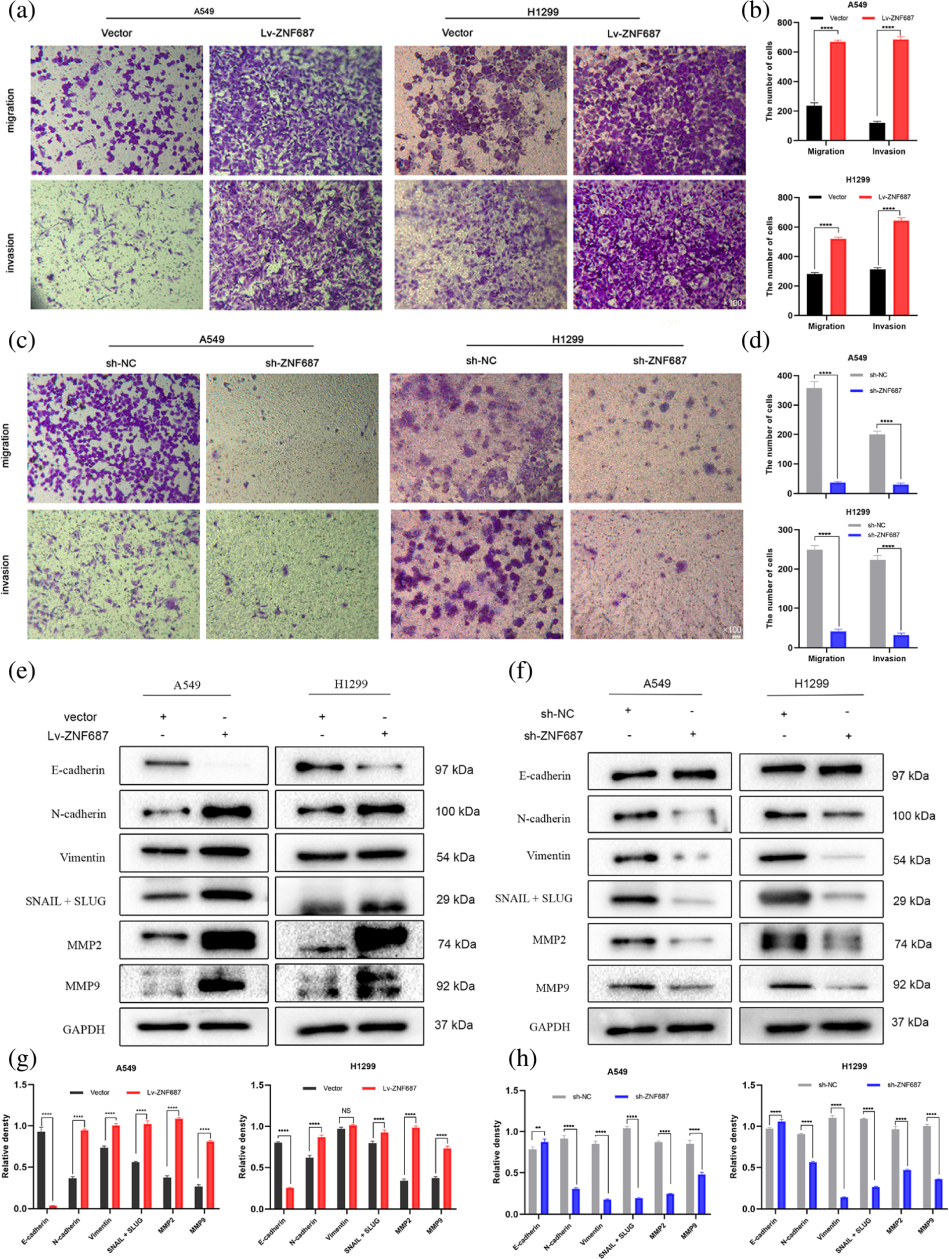
(a–d) A two‐chamber transwell assay was employed to evaluate the migration and invasion of A549 and H1299 cells. (e–h) Western blotting was conducted to determine epithelial‐mesenchymal transition (EMT)‐related protein expression. *p < 0.05; **p < 0.01; ***p < 0.001; and **** *p* < 0.0001.

We examined whether ZNF687 influences LUAD cell migration and invasion. Transwell migration and invasion examinations revealed that ZNF687 knockdown reduced the migration and invasion of A549 and H1299 cells. In contrast, ZNF687 overexpression elevated the migration and invasion capabilities (Figures 5a–d).

Previous studies have shown that epithelial‐mesenchymal transition (EMT) is widely recognized as a critical cancer invasion and metastasis mechanism. The EMT‐related protein expression was identified in our investigation using western blotting. The outcomes exhibited that ZNF687 knockdown reduced the abundance of the mesenchymal markers N‐cadherin, vimentin, Snail, and Slug but raised the abundance of the epithelial marker E‐cadherin in A549 and H1299 cells. As expected, experiments demonstrated that ZNF687 knockdown diminished MMP2 and MMP9 expressions related to invasion and metastasis. In contrast, ZNF687 overexpression demonstrated the opposite effect (Figures 5e–h). These data indicated that ZNF687 overexpression may promote the metastasis of LUAD cells through EMT induction.

### ZNF687 had an oncogenic role in LUAD cells by improving the PI3K/AKT signaling pathway

**FIGURE 6:**
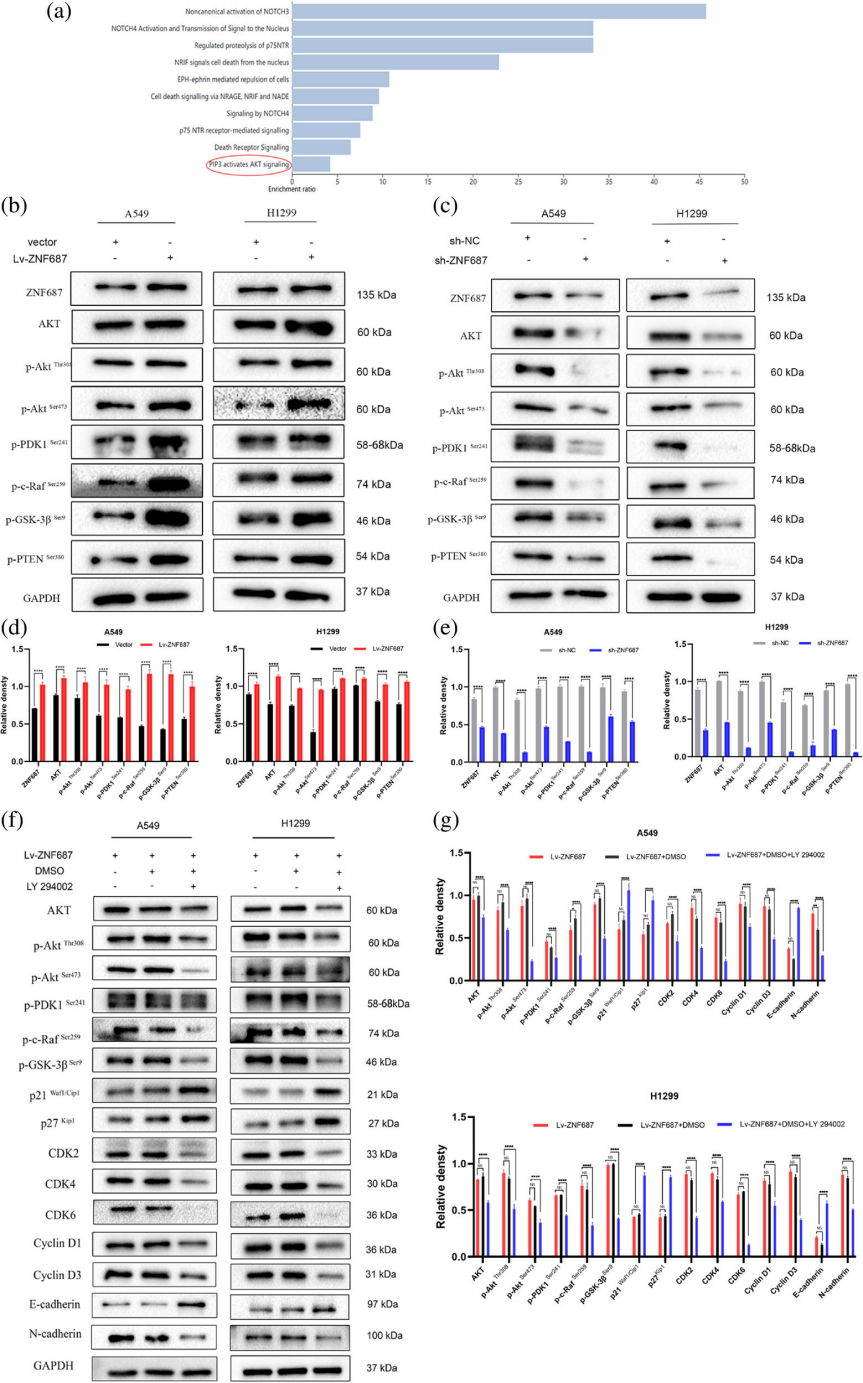
(a) KEGG pathway enrichment analyses of ZNF687‐related genes by WebGestalt database suggested a relation to PIP3 activating the AKT signaling pathway. (b–e) Quantification of AKT, p‐AKT^Thr308^, p‐AKT^Ser473^, p‐PDK1^Ser241^, P‐C‐Raf^Ser259^, p‐GSK‐3β^Ser9^, and p‐PTEN^Ser38^ proteins via western blotting in A549 and H1299 cells. (f, g) Expression of proteins connected to cell growth, migration, and invasion after ZNF687 overexpression was treated with dimethyl sulfoxide (DMSO) or the PI3K inhibitor LY294002 by western blotting. *p < 0.05; **p < 0.01; ***p < 0.001; **** *p* < 0.0001. ns, nonsignificant.

A bioinformatic study was performed to further discover the regulatory pathway of the downstream signaling pathway of ZNF687. The top 100 ZNF687‐correlated genes from LUAD TCGA tumor and normal tissues were acquired from the GEPIA2 database and KEGG pathway enrichment analyses of ZNF687‐related genes from the WebGestalt database. The results suggested that KEGG pathway enrichment analyses were related to cell death signaling, the activation NOTCH3 and NOTCH4 and signal transmission to the nucleus, and the PIP3 activating AKT signaling pathway (Figure 6a). Due to the fact that the PI3K/AKT mechanism regulates metastasis and is frequently activated in LUAD, combined with the WebGestalt bioinformatic analysis results, whether the PI3K/PIP3/AKT mechanism has a function in ZNF687‐related LUAD progression was investigated.

In our study, the outcomes of western blotting analyses revealed that ZNF687 knockdown reduced the levels of AKT, p‐AKT^Thr308^, p‐AKT^Ser473^, p‐PDK1^Ser241^, P‐C‐Raf^Ser259^, p‐GSK‐3β^Ser9^, and p‐PTEN^Ser38^. However, ZNF687 overexpression enhanced the phosphorylation of these kinases (Figure 6b–e).

To further validate that ZNF687 impacts PI3K/AKT signaling, overexpression of ZNF687 cells were supplemented with LY294002, a PI3K suppressor. Our rescue experiments showed that LY294002 partially restored p21 ^Waf1/Cip1^, p27 ^Kip1^, and E‐cadherin expression and decreased AKT, p‐AKT^Thr308^, p‐AKT^Ser473^, p‐PDK1^Ser241^, P‐C‐Raf^Ser259^, p‐GSK‐3β^Ser9^, p‐PTEN^Ser380^, CDK2, CDK4, CDK6, cyclin D1, cyclin D3, and N‐cadherin levels in A549 and H1299 cells (Figure 6f,g). These findings revealed that ZNF687 may have an oncogenic role by activating the PI3K/AKT mechanism to enhance EMT and regulate cell cycle development in LUAD cells.

**FIGURE 7:**
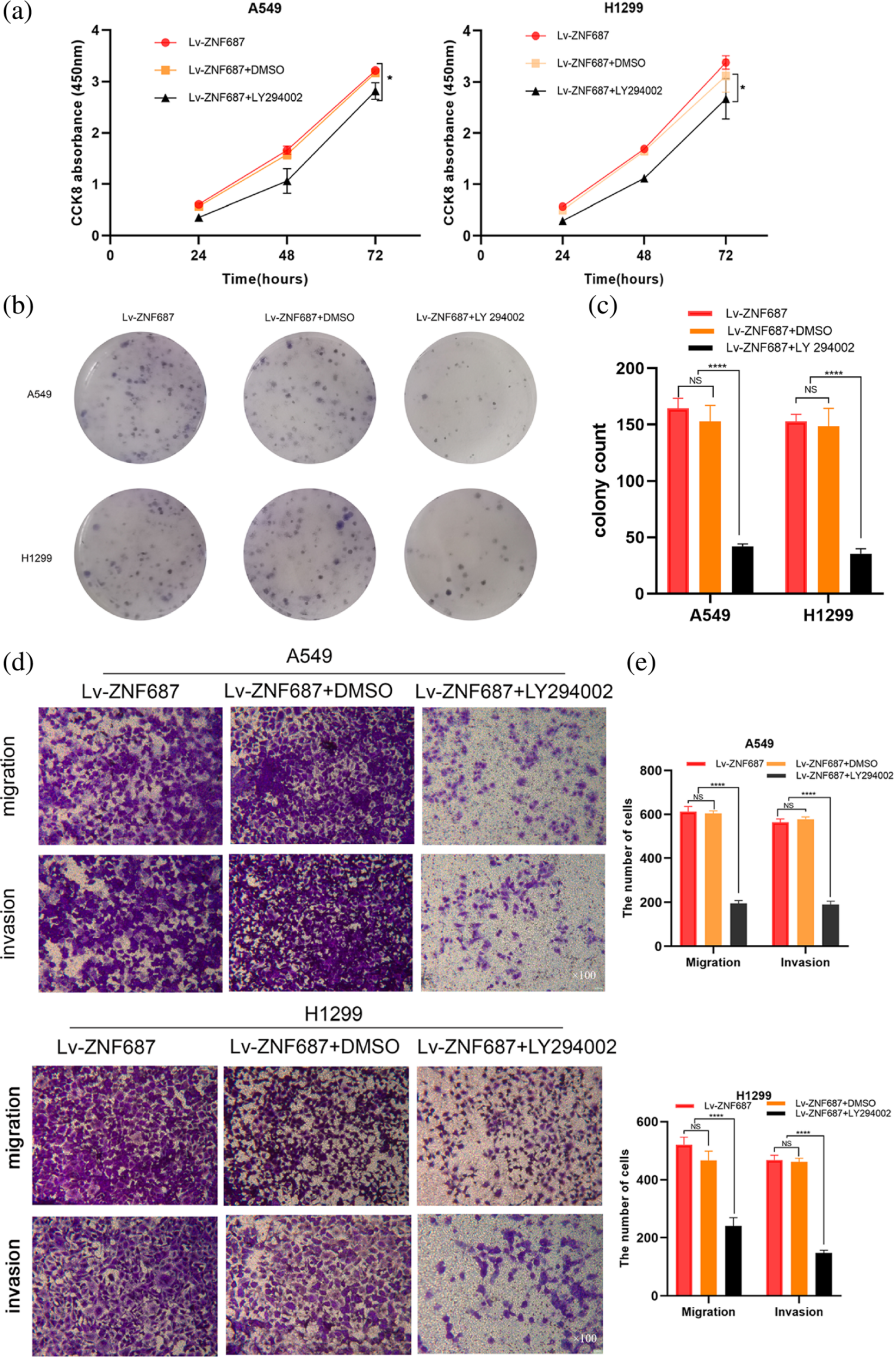
The rescue experiments evaluated the cell growth, migration, and invasion abilities of LY294002 efficacy in the overexpression of ZNF687 cells using (a) the cell counting kit‐8 (CCK‐8) assay, (b, c) the colony formation assay and (d, e) transwell migration and invasion assays, respectively. *p < 0.05; ** *p* < 0.01; *** *p* < 0.001; and**** *p* < 0.0001. ns, nonsignificant.

In the rescue experiments, the CCK‐8 and colony formation assays revealed that LY294002 inhibited the enhanced growth capability of LUAD cells caused by ZNF687 overexpression (Figure 7a, b, c). We also detected that LY294002 therapy abolished the improved migration and invasion induced by ZNF687 overexpression (Figure 7d, e).

These findings demonstrated that ZNF687 may serve in enhancing LUAD cell growth and tumor progression by stimulating the PI3K/AKT signaling mechanism.

## DISCUSSION

Zinc finger proteins comprise the prominent transcription factor family in higher vertebrates. Zinc finger proteins can be separated into eight groups based on combinations of the conserved amino acid residues Cys and His binding to zinc ions, including C2H2‐like zinc finger ([C2H2] like). Zinc finger proteins have a significant function in malignant tumors.^18^, ^19^ ZNF687 is a newly defined C2H2 zinc finger protein. According to recent findings, acute myeloid leukemia and giant cell cancers of the bone exhibit overexpressed and mutant ZNF687.^7^, ^8^ ZNF687 plays a crucial role in enhancing tumorigenesis and controlling stem cell‐like features in HCC. The mechanism is related to the regulation of SOX2, NANOG, and OCT4.^10^ Herein, we determined that ZNF687 was highly expressed in LUAD tissues, which was discovered using western blotting and IHC. This study demonstrated that high ZNF687 expression may act as an important biomarker for poor prognosis of patients with LUAD.

P21(WAF1/CIP1; CDKN1a) is a worldwide cell cycle suppressor in which p53 plays a crucial role either directly or indirectly.^20^ P21 binds to, and suppresses, the cyclin CDK2, CDK1, and CDK4,6 complexes, thereby inhibiting cell cycle development in the G1 and S phases.^21^, ^22^ P21 is a dual regulator influenced by the genotoxic stress types and levels, cell type, cellular localization, and p53 status.^23^, ^24^ A previous bioinformatic study revealed that ZNF687 may be considered a potential p21 suppressor gene; however, the mechanism remains unclear.^17^ In our study, ZNF687 knockdown inhibited the G1‐S transition in flow cytometry assays. In contrast, ZNF687 overexpression enhanced the G1‐S phase transition. To further explore the mechanisms behind this regulation, qRT–PCR and western blotting experiments were conducted. ZNF687 overexpression increased the mRNA and protein levels of CDK2, CDK4, CDK6, cyclin D1, and cyclin D3 but reduced p53, p21, and p27 levels, thus demonstrating that G1/S phase development was stimulated. Conversely, ZNF687 knockdown was observed to have the opposite effect. Therefore, we suggest that ZNF687 may regulate G1/S cell cycle progression by downregulating p21.

EMT is a cellular program related to malignant development, wound healing, and embryogenesis. In cancers, EMT is associated with enhanced tumorigenesis, invasion, metastasis, and treatment resistance.^25^, ^26^, ^27^ Western blotting analysis conducted in our investigation revealed that ZNF687 knockdown reduced the amount of the mesenchymal biomarkers N‐cadherin, vimentin, Snail, and Slug while increasing the abundance of the epithelial biomarker E‐cadherin. However, ZNF687 overexpression had the opposite impact. The expression and activation of matrix metalloproteinases (MMPs) are increased in cancers, and EMT occurrence is positively correlated with the levels of MMP2 and MMP9.^28^ Herein, we experimentally revealed that ZNF687 overexpression enhanced the MMP2 and MMP9 expressions, suggesting that ZNF687 may affect the invasion and migration of LUAD through EMT.

However, it is unclear which signaling pathway ZNF687 employs to regulate the cell cycle and EMT in order to participate in the growth, invasion, and migration of LUAD. This is an issue that has not yet been answered. Using bioinformatic analysis, we found that ZNF687 was related to the PIP3‐activated AKT signaling pathway, a pathway frequently involved in human cancers, particularly lung cancer.^29^, ^30^ Moreover, it has a crucial function in suppressing apoptosis, sustaining angiogenesis, resisting growth signals, and promoting cell growth by affecting the activation state of numerous downstream effector molecules.^31^ In our study, we demonstrated that ZNF687 knockdown reduced AKT, p‐AKT^Thr308^, p‐AKT^Ser473^, p‐PDK1^Ser241^, P‐C‐Raf^Ser259^, p‐GSK‐3β^Ser9^, and p‐PTEN^Ser380^. However, upregulated ZNF687 enhanced the phosphorylation of these kinases. To determine whether ZNF687 affects LUAD cells via PI3K/AKT signaling pathway, we treated cells overexpressing ZNF687 with the PI3K inhibitor LY294002. Subsequently, rescue experiments indicated that LY294002 inhibited and attenuated the cancer promotion impact via ZNF687 overexpression.

Regarding p21 regulation, the PI3K/AKT signaling pathway has a vital function,^32^, ^33^, ^34^ and we initially explored whether ZNF687 regulates p21 through the PI3K/AKT signaling mechanism. In our investigation, PI3K/AKT signaling‐related molecules and p21‐related target genes were activated after ZNF687 enhancement. LY294002 also altered p21 cell cycle‐related molecules and attenuated the G1‐S transition induced by ZNF687 overexpression. Therefore, we suggest that ZNF687 may enhanced the cell cycle transition from G0/G1 to S phase by modulating the PI3K/AKT/p21 signaling pathway and promoting LUAD growth.

PI3K/AKT signaling has been identified to enhance EMT and tumor development in several studies.^35^, ^36^ GSK‐3β, a multifunctional serine/threonine kinase, is crucial in the modulation of glycogen metabolism. It is a downstream gene of the PI3K/AKT signaling pathway and may phosphorylate the Snail transcription factor to modulate EMT.^37^ Our previous study confirmed that PI3K/AKT/GSK‐3β can regulate EMT.^38^ In this study, we found that EMT‐related molecules were altered following the stimulation of the PI3K/AKT signaling mechanism. The results proved that LY294002 restored E‐cadherin expression and decreased N‐cadherin levels. Our findings suggest that ZNF687 may regulate EMT by regulating the Snail transcription factor through the PI3K/AKT/GSK‐3β signaling mechanism, thereby participating in LUAD cell invasion and metastasis.

Although we discovered that ZNF687 overexpression may trigger the malignant phenotype of LUAD, there were a number of limitations established in this study. First, only a PI3K inhibitor was added to ZNF687 overexpression in the PI3K/AKT signaling mechanism recovery experiment, and a PI3K agonist was not added to ZNF687 knockdown cells to validate the PI3K/AKT signaling mechanism. Second, no in vivo animal trials were conducted; the experiment was only validated in vitro. Third, there may be other regulatory mechanisms of ZNF687 in the LUAD cell cycle and signaling pathways. These issues will continue to be investigated in subsequent experiments.

**FIGURE 8:**
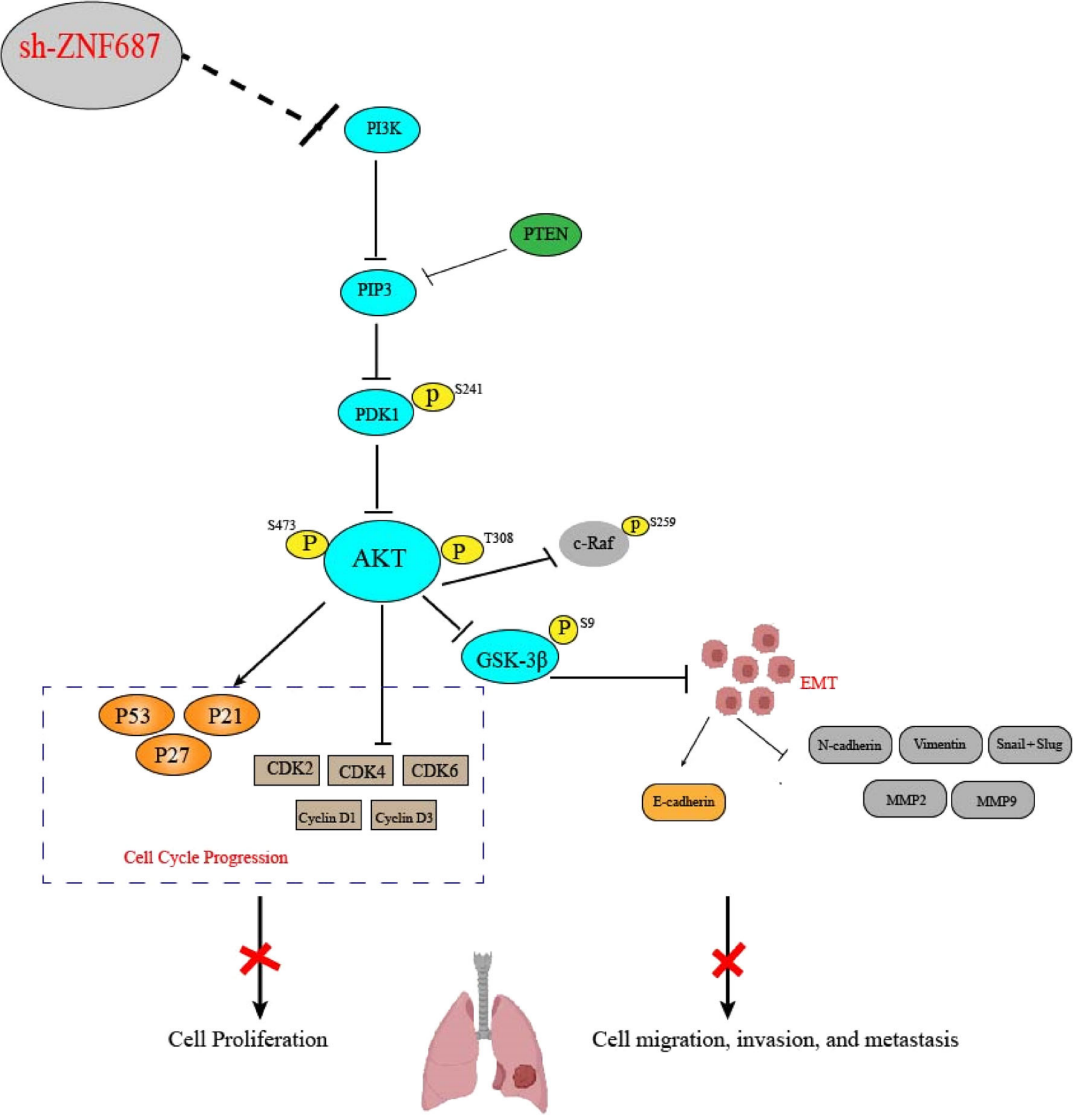
By suppressing the PI3K/AKT signaling pathway, ZNF687 knockdown may lower the growth, invasion, and migration of lung adenocarcinoma (LUAD) cells.

In conclusion, our study proved that ZNF687 is highly expressed in LUAD and associated with a poor prognosis. We also showed that ZNF687 overexpression may promote cell migration, invasion, and EMT by modulating the PI3K/AKT signaling pathway. It may also enhance the G1/S phase cell cycle transition and cell growth. Conversely, ZNF687 knockdown decreased LUAD cell proliferation and tumor progression (Figure 8). These outcomes reveal that ZNF687 may possibly be a beneficial predictive biomarker and a therapeutic target in LUAD.

## AUTHOR CONTRIBUTIONS

Study concept and design: MC. Acquisition of data: MCL, XCW, HQS, ZH and XWX. Drafting of the manuscript: MCL. Analysis and interpretation of the data: YML, ZXT, JQY, MHZ and YLL. Critical revision of the manuscript for important intellectual content: ZHL and MC.

## FUNDING INFORMATION

This study was supported by the Natural Science Foundation of Guangdong Province (2019A1515110022), the Medical Research Foundation of Guangdong Province (A2020607), the Science and Technology Project of Jiangxi Provincial Health Commission (220211693), the Science and Technology Project of Jiangxi Provincial Administration of Traditional Chinese Medicine (2022B961), the Science and Technology Project of Ganzhou (GZ2021ZSF029, 2022DSYS9969), the Science and Technology Research Project of Education Department of Jiangxi Province (GJJ2201415) and the Doctoral Research Initiation Project of the First Affiliated Hospital of Gannan Medical University (QD075).

## CONFLICT OF INTEREST STATEMENT

The authors declare no conflicts of interest.

## ETHICS STATEMENT

The tissue microarray was reviewed and approved by the Ethics Committee of Shanghai Outdo Biotech Co., Ltd. (Shanghai, China) (YBM‐05‐02). All of the human genetic specimens were reviewed and approved by the Ethics Committee of the First Affiliated Hospital of Gannan Medical University (LCSC‐2021082603).

## Supporting information


**Data S1:** Supporting Information
