# Supplementary material for: Oncogenic zinc finger protein ZNF687 accelerates lung adenocarcinoma cell proliferation and tumor progression by activating the PI3K/AKT signaling pathway
Source: Thorac Cancer. 2023 Mar 21;14(14):1223–38. doi: 10.1111/1759-7714.14856 (PMC10175037; doi:10.1111/1759-7714.14856)
Supplement: Supplementary file 1 — Data S1: Supporting Information [file TCA-14-1223-s001.docx]

**Oncogenic zinc finger protein ZNF687 accelerates lung adenocarcinoma cell proliferation and tumor progression by activating PI3K/AKT signaling pathway**

**Appendix. Supplementary data**

| Gene name | Primer ID | Primer Sequence (5'->3') |
| --- | --- | --- |
| p53 | p53 F | GCCCATCCTCACCATCATCACAC |
| p53 | p53 R | GCACAAACACGCACCTCAAAGC |
|  |  |  |
| P21 | P21 F | GACTTTGTCACCGAGACACCACTG |
| P21 | P21 R | CCTGCCTCCTCCCAACTCATCC |
|  |  |  |
| P27 | P27 F | GCTTGCCCGAGTTCTACTACAGAC |
| P27 | P27 R | ACCAAATGCGTGTCCTCAGAGTTAG |
|  |  |  |
| CDK2 | CDK2 F | TGCCTGATTACAAGCCAAGTTTCCC |
| CDK2 | CDK2 R | TTGCGATAACAAGCTCCGTCCATC |
|  |  |  |
| CDK4 | CDK4 F | TTGCCAGCCGAAACGATCAAGG |
| CDK4 | CDK4 R | TCCACCACTTGTCACCAGAATGTTC |
|  |  |  |
| CDK6 | CDK6 F | GTGACCAGCAGCGGACAAATAAAAC |
| CDK6 | CDK6 R | ACGACCACTGAGGTTAGAGCCATC |
|  |  |  |
| Cyclin D1 | Cyclin D1 F | GCCCTCGGTGTCCTACTTCAAATG |
| Cyclin D1 | Cyclin D1 R | TCCTCCTCGCACTTCTGTTCCTC |
|  |  |  |
| Cyclin D3 | Cyclin D3 F | CAGGGAGAGCCTCAGGGAAGC |
| Cyclin D3 | Cyclin D3 R | ACATCTGTAGGAGTGCTGGTCTGG |
|  |  |  |
| ZNF687 | ZNF687 F | ACTCAGGTCCCCTCAGATCC |
| ZNF687 | ZNF687 R | TCACCCAACTGTACGCTCAC |
| GAPDH | GAPDH F | CAGGAGGCATTGCTGATGAT |
| GAPDH | GAPDH R | GAAGGCTGGGGCTCATTT |

**The primers are listed in Supplementary Table S1.**

| Target | Source | Catalog No. | Application |
| --- | --- | --- | --- |
| ZNF687 | Invitrogen | A303-278A-T | WB |
| Cell Cycle Regulation Antibody Sampler Kit | Cell Signaling Technology | #9932 | WB |
| Phospho-AKT Pathway Antibody Sampler Kit | Cell Signaling Technology | #9916 | WB |
| EMT Marker /Epithelial to Mesenchymal Transition Marker | abcam | ab216833 | WB |
| MMP2 | abcam | ab92536 | WB |
| MMP9 | abcam | ab76003 | WB |
| GAPDH | abcam | ab181602 | WB |

**The antibodies are listed in Supplementary Table S2.**

The top 100 ZNF687-correlated genes from LUAD TCGA tumor and normal tissues by GEPIA2 database are listed in Supplementary Table S3.

| Gene Symbol | Gene ID | PCC |
| --- | --- | --- |
| SF3B4 | ENSG00000143368.9 | 0.8 |
| TARS2 | ENSG00000143374.14 | 0.79 |
| VPS72 | ENSG00000163159.11 | 0.77 |
| PYGO2 | ENSG00000163348.3 | 0.76 |
| PRUNE | ENSG00000143363.15 | 0.75 |
| RPRD2 | ENSG00000163125.15 | 0.72 |
| TDRKH | ENSG00000182134.15 | 0.71 |
| TUFT1 | ENSG00000143367.15 | 0.71 |
| PRCC | ENSG00000143294.14 | 0.7 |
| PSMD4 | ENSG00000159352.15 | 0.7 |
| APH1A | ENSG00000117362.12 | 0.7 |
| UBE2Q1 | ENSG00000160714.9 | 0.69 |
| BCL9 | ENSG00000116128.9 | 0.69 |
| MRPL9 | ENSG00000143436.10 | 0.68 |
| LYSMD1 | ENSG00000163155.11 | 0.68 |
| CHTOP | ENSG00000160679.12 | 0.67 |
| FLAD1 | ENSG00000160688.18 | 0.67 |
| PSMB4 | ENSG00000159377.10 | 0.66 |
| ZBTB7B | ENSG00000160685.13 | 0.66 |
| C1orf56 | ENSG00000143443.9 | 0.65 |
| EFNA4 | ENSG00000243364.7 | 0.65 |
| ENSA | ENSG00000143420.17 | 0.65 |
| VPS45 | ENSG00000136631.12 | 0.64 |
| SETDB1 | ENSG00000143379.12 | 0.64 |
| ILF2 | ENSG00000143621.16 | 0.62 |
| SCAMP3 | ENSG00000116521.10 | 0.62 |
| SNX27 | ENSG00000143376.12 | 0.61 |
| UBQLN4 | ENSG00000160803.7 | 0.61 |
| ANP32E | ENSG00000143401.14 | 0.61 |
| SCNM1 | ENSG00000163156.11 | 0.61 |
| PI4KB | ENSG00000143393.16 | 0.6 |
| MTX1 | ENSG00000173171.14 | 0.6 |
| RORC | ENSG00000143365.16 | 0.6 |
| COPA | ENSG00000122218.14 | 0.6 |
| POLR3C | ENSG00000186141.8 | 0.6 |
| PMF1 | ENSG00000160783.19 | 0.6 |
| ADAR | ENSG00000160710.15 | 0.59 |
| ISG20L2 | ENSG00000143319.16 | 0.59 |
| SNAP47 | ENSG00000143740.14 | 0.59 |
| RBM8A | ENSG00000265241.6 | 0.59 |
| NCSTN | ENSG00000162736.15 | 0.59 |
| PIGM | ENSG00000143315.6 | 0.58 |
| GOLPH3L | ENSG00000143457.10 | 0.58 |
| ZNF212 | ENSG00000170260.8 | 0.57 |
| DAP3 | ENSG00000132676.15 | 0.57 |
| CCT3 | ENSG00000163468.14 | 0.57 |
| GPR89A | ENSG00000117262.18 | 0.57 |
| KDF1 | ENSG00000175707.8 | 0.57 |
| BOLA1 | ENSG00000178096.8 | 0.57 |
| MRPS21 | ENSG00000266472.5 | 0.56 |
| RUSC1 | ENSG00000160753.15 | 0.56 |
| B4GALT3 | ENSG00000158850.14 | 0.56 |
| HDGF | ENSG00000143321.18 | 0.55 |
| CERS2 | ENSG00000143418.19 | 0.55 |
| OTUD7B | ENSG00000264522.5 | 0.55 |
| UBE2O | ENSG00000175931.12 | 0.55 |
| THEM4 | ENSG00000159445.12 | 0.55 |
| KLHL12 | ENSG00000117153.15 | 0.54 |
| C10orf35 | ENSG00000171224.8 | 0.54 |
| C2CD4D | ENSG00000225556.1 | 0.54 |
| PODXL2 | ENSG00000114631.10 | 0.54 |
| IQGAP3 | ENSG00000183856.10 | 0.54 |
| PARS2 | ENSG00000162396.5 | 0.54 |
| CBX4 | ENSG00000141582.14 | 0.53 |
| FAM189B | ENSG00000160767.20 | 0.53 |
| EHMT2 | ENSG00000204371.11 | 0.53 |
| RAB25 | ENSG00000132698.13 | 0.53 |
| FAM86JP | ENSG00000171084.15 | 0.53 |
| PIP5K1A | ENSG00000143398.19 | 0.53 |
| ZNF786 | ENSG00000197362.13 | 0.53 |
| PARP1 | ENSG00000143799.12 | 0.53 |
| SLC39A1 | ENSG00000143570.17 | 0.53 |
| UBAP2L | ENSG00000143569.18 | 0.53 |
| AL450992.2 | ENSG00000234614.1 | 0.53 |
| ZNF48 | ENSG00000180035.10 | 0.52 |
| SPTBN2 | ENSG00000173898.11 | 0.52 |
| RBBP8NL | ENSG00000130701.3 | 0.52 |
| DUSP12 | ENSG00000081721.11 | 0.52 |
| KIAA1522 | ENSG00000162522.10 | 0.52 |
| PEX11B | ENSG00000131779.10 | 0.52 |
| ZNF574 | ENSG00000105732.11 | 0.52 |
| ARHGEF19 | ENSG00000142632.16 | 0.52 |
| ZBTB42 | ENSG00000179627.9 | 0.52 |
| PRR36 | ENSG00000183248.11 | 0.52 |
| MEX3A | ENSG00000254726.2 | 0.52 |
| RABIF | ENSG00000183155.4 | 0.52 |
| KDM5B | ENSG00000117139.16 | 0.52 |
| ZBTB9 | ENSG00000213588.5 | 0.52 |
| GBA | ENSG00000177628.15 | 0.52 |
| SLC25A44 | ENSG00000160785.13 | 0.52 |
| DOLPP1 | ENSG00000167130.17 | 0.52 |
| CHD1L | ENSG00000131778.17 | 0.52 |
| ARHGEF16 | ENSG00000130762.14 | 0.52 |
| GPR89B | ENSG00000188092.14 | 0.52 |
| FAM134A | ENSG00000144567.10 | 0.52 |
| PFDN2 | ENSG00000143256.4 | 0.52 |
| SOX12 | ENSG00000177732.7 | 0.52 |
| SMYD5 | ENSG00000135632.11 | 0.52 |
| RP5-1061H20.4 | ENSG00000177788.5 | 0.51 |
| LAMTOR2 | ENSG00000116586.11 | 0.51 |
